# Bridging pathogenesis and therapy in focal segmental glomerulosclerosis: the rise of dual receptor blockade with sparsentan

**DOI:** 10.1097/MS9.0000000000005263

**Published:** 2026-06-15

**Authors:** Muhammad Ali Abid, Muhammad Hafi Abid, Abdul Haseeb Hasan, Muhammad Daniyal Afzal

**Affiliations:** a Department of Medicine, King Edward Medical University, Lahore, Pakistan; b Department of Medicine, Faisalabad Medical University, Faisalabad, Pakistan; c Department of Medicine, Kyrgyz State Medical Academy, Bishkek, Kyrgyzstan

**Keywords:** endothelin receptor antagonists, focal segmental glomerulosclerosis, proteinuria, sparsentan

## Abstract

Focal segmental glomerulosclerosis (FSGS) represents a heterogeneous clinicopathological entity characterized by podocyte injury, progressive glomerular scarring, and a high risk of progression to end-stage renal disease. Despite advances in understanding its pathophysiology, therapeutic options have remained limited, largely centered on supportive care and immunosuppression, with inconsistent efficacy and significant adverse effects. The recent approval of sparsentan, a dual endothelin type A and angiotensin II type 1 receptor antagonist, marks a significant advancement in the management of FSGS by targeting key hemodynamic and molecular pathways implicated in disease progression. This article provides a comprehensive overview of the pathogenesis of FSGS, emphasizing the central role of podocyte injury and maladaptive responses to mechanical stress. It further reviews current treatment strategies and examines the clinical evidence underpinning the approval of sparsentan, particularly findings from the DUPLEX trial. Although sparsentan did not demonstrate a statistically significant benefit in preserving estimated glomerular filtration rate, it achieved a clinically meaningful reduction in proteinuria compared with irbesartan. The safety profile was generally acceptable, with manageable adverse effects, including hyperkalemia and hypotension. While sparsentan introduces a novel, non-immunosuppressive therapeutic option, its long-term impact on renal outcomes remains to be fully established. Ongoing research will be critical to defining its role within combination regimens and optimizing patient selection in this complex disease spectrum.

## Introduction

Focal segmental glomerulosclerosis (FSGS) is not a single disease but a histopathological pattern of kidney injury characterized by scarring that affects only parts of some glomeruli, most commonly seen in association with podocyte injury and loss^[^1,2^]^. Podocytes are specialized cells that are essential for the maintenance of the glomerular filtration barrier, and their depletion results in structural damage to the glomerular capillary tuft^[^1^]^. The 2021 Kidney Disease Improving Global Outcomes classification categorizes FSGS into primary (idiopathic) FSGS, genetic FSGS, secondary FSGS, and FSGS of undetermined cause based on clinical features, genetics, etiology, and histopathology to improve diagnostic and clinical utility^[^1^]^. Patients with FSGS present clinically with edema, proteinuria, and progressive worsening of renal function^[^1,3^]^. The global incidence of FSGS is approximately seven cases per one million population, and the prevalence among glomerular diseases is around 4%^[^1^]^. FSGS is a leading cause of end-stage renal disease (ESRD) worldwide^[^1^]^.

## Pathophysiology of FSGS

FSGS is a podocytopathy, which primarily involves injury to the podocytes without any immune complex deposition^[^1^]^. Podocytes are exposed to mechanical stress, including tensile stress from increased capillary pressure and shear stress from increased filtrate flow^[^1,3^]^. This stress can damage their structure, leading to the effacement of foot processes resulting from reorganization of their actin cytoskeleton and loss of slit diaphragm integrity^[^3^]^. This effacement is initially protective, but persistent injury results in depletion of podocytes, a key driver of the disease^[^1,3^]^. The kidney attempts to repair the damage through hypertrophy of remaining podocytes^[^1^]^. When injury continues, areas of the glomerular basement membrane become exposed due to the loss of podocyte coverage^[^1^]^. These exposed areas stick to Bowman’s capsule, forming adhesions^[^1,3^]^. This leads to the collapse of capillary loops and the accumulation of extracellular matrix, which results in segmental scarring that can progress to global glomerulosclerosis^[^1^]^.

## Current therapeutic strategies

The current management of FSGS involves the reduction of proteinuria, a surrogate marker that strongly correlates with improved kidney survival^[^1,2^]^. Supportive treatment is achieved using renin-angiotensin-aldosterone system (RAAS) blockade with angiotensin-converting enzyme inhibitors and angiotensin receptor blockers, along with salt restriction and blood pressure control to reduce intraglomerular pressure and protect podocytes^[^3^]^. Newer agents, such as sodium-glucose cotransporter 2 inhibitors like dapagliflozin and nonsteroidal mineralocorticoid antagonists, show favorable results, but they are not formally indicated for FSGS, making their use off-label and still controversial^[^1^]^. For the management of primary FSGS, immunosuppression using glucocorticoids is employed, with calcineurin inhibitors (CNIs) such as cyclosporine or tacrolimus being used in cases of steroid resistance^[^1,2^]^. The use of immunosuppressants is not recommended in secondary FSGS, genetic FSGS, and FSGS of undetermined cause, where the removal of the offending agent or drug and treatment of the underlying cause, along with supportive care, is the mainstay of management^[^1,2^]^.

## Sparsentan: Mechanism of action and dosing

The U.S. Food and Drug Administration (FDA) has recently approved the use of FILSPARI^TM^ (sparsentan) oral tablets for the management of FSGS in patients 8 years and older, making it the first drug to be approved for the treatment of FSGS^[^4^]^. For FSGS, sparsentan dosage is weight-dependent^[^4^]^. A once-daily dose of 400 mg is initiated in patients weighing >50 kg, which is increased to 800 mg once daily after 14 days as tolerated^[^4^]^. Patients weighing ≤50 kg are started at a once-daily dose of 200 mg, which is increased to a 400 mg once-daily dose as tolerated^[^4^]^. Discontinuation of RAAS inhibitors and endothelin receptor blockers is required before initiation of treatment, and re-titration is required if treatment is restarted after discontinuation^[^4^]^.

Sparsentan is a dual endothelin and angiotensin II receptor antagonist ^[^4,5^]^. It blocks the two key receptors involved in the pathogenesis of FSGS: the endothelin type A (ETA) receptor and the angiotensin II type 1 (AT1) receptor^[^5^]^. By antagonizing the AT1 receptor, sparsentan decreases the vasoconstriction, inflammation, and proteinuria caused by angiotensin II^[^5^]^. By blocking the ETA receptor, sparsentan reduces the vasoconstrictive action of endothelin-1, a potent vasoconstrictor often found elevated in chronic kidney disease. ETA receptor activation contributes to kidney injury by reducing blood flow to the kidneys and promoting proteinuria; its blockade helps mitigate these effects^[^5^]^. Sparsentan, through this dual blockade of the ETA receptor and AT1 receptor, targets two pathways responsible for causing proteinuria^[^5^]^.

## Clinical evidence: The DUPLEX TRIAL

The approval was based on results from a randomized, double-blinded, active-controlled portion of a multicenter, global trial DUPLEX (NCT03493685)^[^4,6^]^. The study included 371 adults and pediatric patients 9 years of age or older with biopsy-proven FSGS or a genetic mutation known to cause FSGS, urine protein-creatinine ratio (UPCR) ≥1.5 g/g, and an estimated glomerular filtration rate (eGFR) ≥30 mL/min/1.73 m^2[^4,6^]^. The patients were randomized into two groups to receive either sparsentan or irbesartan for a duration of 108 weeks^[^4,6^]^. The mean age of participants at the time of enrollment was 42 years, and the study included a total of 35 pediatric patients^[^4,6^]^. The mean eGFR taken at the time of enrollment was 63 mL/min/1.73 m^2,^ and the mean UPCR was 3.1 g/g in both treatment groups^[^4,6^]^. The study did not include patients with secondary FSGS or those who were recipients of kidney transplants^[^4,6^]^. Patients who had received potent immunosuppressive therapies such as rituximab, cyclophosphamide, or abatacept within 3 months prior to screening were excluded^[^4,6^]^. Other immunosuppressive agents, including corticosteroids, CNIs, mycophenolate mofetil, and azathioprine, were permitted only if patients were on a stable dose for at least 1 month before enrollment^[^4,6^]^. Concurrent use of RAAS inhibitors, endothelin receptor antagonists, and potassium-sparing diuretics was prohibited^[^4,6^]^. Individuals on RAAS inhibitors were required to undergo a 2-week washout before randomization^[^4,6^]^.

The primary endpoint at the final analysis was the rate of change in eGFR from baseline at week 108^[^4,6^]^. The primary efficacy endpoint did not reach statistical significance^[^4^]^. In the overall DUPLEX study population, the adjusted mean change in eGFR from baseline to week 108 was −14.3 mL/min/1.73 m^2^ with sparsentan, in contrast to −13.3 mL/min/1.73 m^2^ with irbesartan^[^4,6^]^. The difference between the two groups was −1.0 mL/min/1.73 m^2^ (95% CI: −4.9 to 2.8), which was not significant^[^4,6^]^. However, sparsentan showed better reduction in proteinuria; a 46% decrease in UPCR from baseline was seen by week 108 compared to a 30% decrease in UPCR from baseline with irbesartan, giving a 22% greater reduction (95% CI: 2%–38%) in favor of sparsentan^[^4,6^]^. Four weeks after stopping study treatment, that is, at week 112, there was no difference between observed eGFR and UPCR changes in the two arms^[^4,6^]^. In patients without nephrotic syndrome, sparsentan achieved a greater UPCR reduction, with a reduction of 48% compared to 27% seen with irbesartan, representing a 29% greater reduction (95% CI: 9%–44%)^[^4,6^]^. In patients with nephrotic syndrome, no difference in UPCR reduction between sparsentan and irbesartan was observed^[^4,6^]^. The effect of treatment on UPCR reduction was broadly consistent across subgroups defined by age, sex, race, and ethnicity^[^4,6^]^.

## Safety profile and adverse effects

Sparsentan was generally well tolerated in patients, with the most common adverse reactions–those occurring in ≥5% of patients–being peripheral edema, hypotension, hyperkalemia, dizziness, and anemia^[^4,6^]^. Key safety considerations include an increased risk of hyperkalemia, especially when used with other agents that raise serum potassium, such as potassium-sparing diuretics. It can also cause hypotension and acute kidney injury^[^4,6^]^. Patients should be monitored for worsening renal function, particularly if they are taking nonsteroidal anti-inflammatory drugs^[^4,6^]^. Sparsentan has several important drug interactions; strong CYP3A inhibitors and inducers should be avoided, while moderate CYP3A inhibitors require monitoring^[^4,6^]^.

## Discussion and future directions

The approval of sparsentan for FSGS represents a fundamental change in the management of FSGS, being the first and only FDA-approved drug specifically indicated for the management of FSGS^[^4^]^. It provides a non-immunosuppressive, orally administered option for FSGS management. Although sparsentan failed to reach statistical significance in the primary efficacy endpoint of reducing eGFR, its main clinical benefit is the meaningful reduction in proteinuria, an important marker for long-term renal outcomes in FSGS^[^1,4^]^. However, this medication is not indicated in cases where the patient has nephrotic syndrome, and the long-term consequences of this drug have not yet been established in terms of serious outcomes like ESRD. The ultimate fate of sparsentan as a treatment modality in patients with FSGS will depend on clinical studies that highlight the importance of prolonged renal preservation and help elucidate its role in combination therapy. In addition, there will always be interest in whether sparsentan, as a dual pathway inhibitor, will compete with or complement other newer therapeutic strategies aimed at targeting podocytes and immune modulation. In practice, careful patient selection, laboratory surveillance, and close monitoring of side effects will still be crucial moving forward.

## Data Availability

Not applicable to this study.

## Research registration unique identification number (UIN)

Not applicable to this study.
